# Extracting semantically enriched events from biomedical literature

**DOI:** 10.1186/1471-2105-13-108

**Published:** 2012-05-23

**Authors:** Makoto Miwa, Paul Thompson, John McNaught, Douglas B Kell, Sophia Ananiadou

**Affiliations:** 1 The National Centre for Text Mining, Manchester Interdisciplinary Biocentre, University of Manchester, 131 Princess Street, Manchester, M1 7DN, UK; 2 School of Computer Science and the Manchester Interdisciplinary Biocentre, University of Manchester, Manchester, M1 7DN, UK; 3 School of Chemistry and the Manchester Interdisciplinary Biocentre, University of Manchester, 131 Princess Street, Manchester, M1 7DN, UK

## Abstract

**Background:**

Research into event-based text mining from the biomedical literature has been growing in popularity to facilitate the development of advanced biomedical text mining systems. Such technology permits advanced search, which goes beyond document or sentence-based retrieval. However, existing event-based systems typically ignore additional information within the textual context of events that can determine, amongst other things, whether an event represents a fact, hypothesis, experimental result or analysis of results, whether it describes new or previously reported knowledge, and whether it is speculated or negated. We refer to such contextual information as *meta-knowledge*. The automatic recognition of such information can permit the training of systems allowing finer-grained searching of events according to the meta-knowledge that is associated with them.

**Results:**

Based on a corpus of 1,000 MEDLINE abstracts, fully manually annotated with both events and associated meta-knowledge, we have constructed a machine learning-based system that automatically assigns meta-knowledge information to events. This system has been integrated into EventMine, a state-of-the-art event extraction system, in order to create a more advanced system (EventMine-MK) that not only extracts events from text automatically, but also assigns five different types of meta-knowledge to these events. The meta-knowledge assignment module of EventMine-MK performs with macro-averaged F-scores in the range of 57-87% on the BioNLP’09 Shared Task corpus. EventMine-MK has been evaluated on the BioNLP’09 Shared Task subtask of detecting negated and speculated events. Our results show that EventMine-MK can outperform other state-of-the-art systems that participated in this task.

**Conclusions:**

We have constructed the first practical system that extracts both events and associated, detailed meta-knowledge information from biomedical literature. The automatically assigned meta-knowledge information can be used to refine search systems, in order to provide an extra search layer beyond entities and assertions, dealing with phenomena such as rhetorical intent, speculations, contradictions and negations. This finer grained search functionality can assist in several important tasks, e.g., database curation (by locating new experimental knowledge) and pathway enrichment (by providing information for inference). To allow easy integration into text mining systems, EventMine-MK is provided as a UIMA component that can be used in the interoperable text mining infrastructure, U-Compare.

## Background

Biomedical text mining [1-3] has focussed largely on recognising relevant biomedical entities and binary relations between these entities (e.g., protein-protein interactions [4,5], gene-disease associations [6,7], etc.). However, the extraction of biomedical events from the literature has been a recent focus of research into biomedical natural language processing, since events are crucial for understanding biomedical processes and functions [3]. Events constitute structured representations of biomedical knowledge. They are usually organised around verbs (e.g., *activate, inhibit*) or nominalised verbs (e.g., *expression*), which we call *trigger expressions*. Events have arguments, which contribute towards the description of the event. These arguments, which can either be entities (e.g., *p53*) or other events, are often assigned semantic roles, which characterise the contribution of the argument to the description of the event. Typical examples of semantic roles include *Cause* (what is responsible for the event occurring) and *Theme* (what is affected by the event occurring). The following sentence serves to exemplify the general format of events: 

· *In addition, it has been established that tumor necrosis factor-alpha (TNF-alpha) can activate the expression of wild type p53 in concert with the nuclear transcription factor, NF-kappa B.* (PMID: 10416957 [8])

Sentence (i) is annotated with 2 events in the BioNLP’09 Shared Task corpus [9]. 

· Event ID: E1, Type: Gene expression, Trigger: *expression*, Theme: *p53*

· Event ID: E2, Type: Positive regulation, Trigger: *activate*, Cause: *tumor necrosis factor-alpha (TNF-alpha)*, Theme: E1

The details provided in events are vital for the development of advanced semantic search applications that allow search criteria to be specified in terms of constraints on these structured events, rather than using the more traditional keywords. However, the textual context of events often provides important information about how they are to be interpreted. We term such information *meta-knowledge*[10]. In most of the existing corpora used for biomedical text mining, such information is either not encoded at all in the event annotations, or otherwise only limited information is present. For example, the BioInfer [11] corpus annotates negated events, whilst the GENIA event corpus [12] and the two BioNLP shared task corpora [9,13] include both negation and basic speculation information. In the shared task corpora, speculation annotation is limited to a simple binary distinction between speculated and non-speculated events. Events in the GENIA event corpus are annotated according to 3 different levels of certainty, but the meaning of each level is not clearly defined. Although the basic meta-knowledge annotation in these corpora allows some useful distinctions between events to be identified, it is not sufficient to distinguish between events that express the following types of information [10,14]: 

· Accepted facts vs. experimental findings.

· Hypotheses vs. interpretations of experimental results.

· Previously reported findings vs. new findings.

The identification of such types of information is only possible if different types of contextual information are considered, e.g., whether the knowledge expressed by events is supported through experimental evidence, or through the citing of references.

The identification of event meta-knowledge can support various domain-specific applications, such as pathway construction and deep semantic search, to satisfy a variety of information needs [14]. This leads to a finer grained level of information extraction, in which papers can be analysed from several different viewpoints. The extraction of fine-grained information is useful not only for biomedical text mining, but also for semantic publishing applications [15-18].

In response to the benefits of recognising event meta-knowledge, as outlined above, we have built an integrated system that automatically extracts biomedical events from the literature, and determines associated meta-knowledge, using machine learning methods. The new system, called EventMine-MK, is an extension of EventMine [19], a machine learning-based state-of-the-art event extraction system that aims to extract structured events with explicit links between triggers and arguments. The EventMine-MK system was trained on the version of the GENIA event corpus [12] that has been enriched with detailed meta-knowledge [10] (henceforth referred to as “the GENIA-MK corpus”).

We chose to use the GENIA-MK corpus for training for a number of reasons. Firstly, in contrast to other event corpora that include only limited information about the interpretation of events, the GENIA-MK corpus includes detailed meta-knowledge annotation for each of the 36,858 events in the corpus. The annotation consists of five different dimensions, each encoding a different aspect of event interpretation, i.e., *Knowledge TypeCertainty LevelPolarityManner* and *Source*[15,20,21]. Each dimension has a fixed set of values, which are clearly defined in the annotation guidelines. Secondly, this corpus is by far the largest of the all of the event corpora. Thirdly, other event corpora generally have larger numbers of event and entity types, which can make the event extraction process more difficult. Since the focus of our work is on the ability of our integrated EventMine-MK system to assign detailed meta-knowledge to extracted events, we wanted to avoid adding extra complexity to the event extraction task.

The EventMine-MK system was constructed in two stages. Firstly, we trained a system to assign meta-knowledge to pre-annotated biomedical events in the GENIA-MK corpus. Intrinsic, stand-alone evaluation of this initial system reveals that its performance in assigning values of different meta-knowledge dimensions to events ranges between 59.2% and 79.7% (macro-averaged F-scores; F-score is a harmonic mean of precision and recall). Compared to a baseline in which the most commonly annotated value in each dimension is assigned, our system shows encouraging performance, with improvements in F-score over the baseline ranging between 8.5% and 49.6%, according to the meta-knowledge dimension under consideration. Secondly, we integrated the meta-knowledge assignment system with EventMine. Thus augmented, EventMine-MK can perform both automatic extraction of events from the literature and automatic assignment of meta-knowledge to these extracted events. Since detailed meta-knowledge annotation at the event level is a new concept, and the GENIA-MK corpus has only recently been released, our system is the first that is able to assign 5 different dimensions of meta-knowledge to events. However, in order to allow comparison with other systems, we have evaluated part of the functionality of EventMine-MK, by applying it to the task of extracting negated and speculated events, which was a subtask of the BioNLP’09 Shared Task (BioNLP’09 ST) [9]. Our results demonstrate that EventMine-MK outperforms other state-of-the-art systems that participated in this task. In order to make it simple to integrate EventMine-MK with larger text mining systems, it has been made available as a UIMA component within the U-Compare interoperable text mining platform [22], which allows the construction of text mining workflows via a drag-and-drop interface.

### Event extraction

Research into the automatic extraction of events has been stimulated by two shared tasks (STs) focussing on event extraction (i.e., BioNLP’09 ST [9] and the BioNLP Shared Task 2011 (BioNLP ST’11) [13,23]). As a result of these shared tasks, several new state-of the-art event extraction systems have been reported that can extract core events, such as *BindingRegulation*, etc., from the BioNLP’09 ST corpus (henceforth referred to as “the ST corpus”) and the GENIA event extraction task corpus from BioNLP-ST’11 (henceforth referred to as “the BioNLP-ST’11 GENIA corpus”) with F-scores greater than 55% (e.g., [24,25]).

#### Event corpora

**Figure 1:**
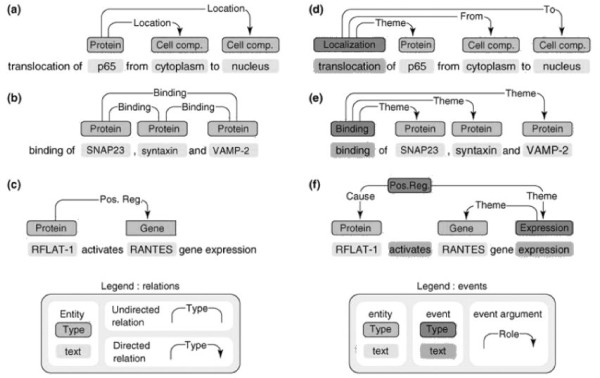
**Relation (left) and event (right) representations.** Three sentences are shown. Each sentence covers a different type of relation/event. Relation annotations for the three sentences are shown on the left hand side, while event annotations are shown on the right-hand side. Examples **a)** and **d)** concern *Localization*, **b)** and **e)** concern *Binding*, and **c)** and **f)** concern *Regulation*.

Figure 1 illustrates relations and events related to one or more entities [3]. A relation is typically represented by a pair of bio-entities, proteins, diseases, etc. Example (a) in Figure 1 illustrates two such relations that involve the entity *p65*. This entity is in *Location* relations with both the entity *cytoplasm* and the entity *nucleus*. Example (d) in Figure 1 shows the same sentence with event annotation. There is a single *Localization* event that involves all three entities. The event expresses the fact that *p65* localises from *cytoplasm* to *nucleus*. Thus, the use of events rather than relations allows for a more detailed encoding of the knowledge that is expressed in texts. The exact representation of events is flexible, and can be adapted to specific user needs. A further type of structured representation of knowledge in text that is somewhat comparable to events is the “nano-publication” [17,18]. Nano-publications are based on linked data/RDF, and consist only of simple subject-predicate-object triples. Thus, compared to events, their expressiveness and flexibility is limited.

Both the BioNLP’09 ST and the BioNLP ST’11 provided corpora with gold-standard event annotations. These can be used to train systems, as well as acting as benchmarks for the evaluation of new systems. For instance, the ST corpus consists of 1,210 MEDLINE abstracts and covers 13,588 biomolecular events involving genes or proteins. This corpus was generated by extracting and reorganising a subset of the events and named entities (NEs) contained within the GENIA event corpus [12], in order to create a more tractable resource on which event extraction systems can be trained. Table 1 summarises the event types and their arguments in the ST corpus. The corpus consists of 9 different types of events, including five simple event types (*Gene ExpressionTranscriptionProtein metabolismPhosphorylation* and *Localization*), each of which takes one core Theme argument, a multi-participant binding event (*Binding*) and three regulation events (*RegulationPositive regulation* and *Negative regulation*). The regulation events are used to capture both biological regulation and general causation. Examples (d), (e) and (f) in Figure 1 illustrate simple, binding and regulation events, respectively. The participants of both simple and binding events are specified to be of the general *Protein* type, while regulation-type events can also take other events as arguments, creating complex event structures. Some of these events take secondary arguments representing locations or sites. Events in the ST corpus are also annotated for negation and speculation.

**Table 1:** Event types and their arguments in the ST corpus

**Event type**	**Primary arguments**	**Secondary arguments**
Gene expression	Theme (P)	
Transcription	Theme (P)	
Protein Catabolism	Theme (P)	
Phosphorylation	Theme (P)	Site
Localization	Theme (P)	AtLoc, ToLoc
Binding	Theme (P)+	Site+
Regulation	Theme(P/Ev), Cause(P/Ev)	Site, CSite
Positive regulation	Theme(P/Ev), Cause(P/Ev)	Site, CSite
Negative regulation	Theme(P/Ev), Cause(P/Ev)	Site, CSite

For each event, argument roles (Theme, Cause) and argument types (P:Protein, Ev:Event) are shown Secondary arguments are optional, and they include AtLoc (the source of an event), ToLoc (the goal or destination of an event), Site (specific domains or regions corresponding to the theme of the event), CSite (specific domains of regions corresponding to the cause of an event). ***Binding*** events can take an arbitrary number of proteins as their theme, and ***Regulation*** events can take events or proteins as their arguments.

In addition to the GENIA event corpus and the corpora from the two BioNLP STs, a number of other gold-standard annotated corpora of biomedical events have been produced by different research groups; each corpus varies in terms of target domain, size, types of events identified, and types and numbers of arguments annotated. BioInfer, the GENIA event corpus and GREC [26] all share similar event representations to the one illustrated above, although there is a substantial difference in their sizes: the GENIA event corpus is the largest, containing containing 1,000 abstracts, GREC contains 240 abstracts, while BioInfer contains 1,110 sentences. The representations of the GeneReg [27] and Angiogenesis Bio-Process [28] corpora are somewhat different. GeneReg, which consists of 314 abstracts, does not annotate explicit links between event triggers and arguments, while the Angiogenesis Bio-Process corpus (262 abstracts) annotates events as single spans containing both triggers and arguments, without identifying the internal structure of the events.

#### Event extraction systems

A large number of event extraction systems have been reported, both as part of the two BioNLP STs described above, and subsequently. The STs provided the first stimulus for the development of systems to extract the types of structured events described above. The functionality of these systems is in contrast to the simpler task of extracting pairs of interacting proteins, which have been covered by other challenges, such as the BioCreative challenges [29-32] and LLL05 [33]. The BioNLP STs have provided standard evaluation benchmarks, including task definitions, data sets for training and tools for evaluation. The STs defined three subtasks: finding basic events with the core arguments *Theme* and *Cause* (Task 1), and attaching to these basic events either secondary arguments, such as location and site (Task 2), or negation and speculation (Task 3). Most systems participated only in Task 1, and fewer than 10 systems tackled Task 2 and/or Task 3 (presumably because of their complexities).

Event extraction systems participating in the BioNLP STs have mostly employed pipeline-based approaches, using machine learning methods based on parse results [19,34-39]. In [34], a pipeline-based system was built to extract events. The system firstly detected event triggers and arguments sequentially, using multi-class support vector machines (SVM), and it subsequently constructed multi-argument events using hand-crafted rules. It achieved the best performance in the BioNLP’09 ST. The second best system [35] in the BioNLP’09 ST detected triggers using dictionaries, and identified arguments through graph kernel-based classification on trimmed dependency graphs, which are built by removing irrelevant lexical information from dependency parse results and adding conceptual class information to the results. Such trimming is useful for detecting events, but it can lead to loss of the information required to calculate meta-knowledge, e.g., it prunes modal verbs such as *may*. A dependency parsing approach to event extraction was proposed in [39]. They detected triggers with a maximum entropy classifier (ME), and then found arguments by applying syntactic parsing technologies (maximum-spanning tree parsing and parse reranking) to pseudo syntactic structures converted from event structures. In [19], the event extraction system EventMine was proposed, which extended the approach introduced in [34] by extending the features and utilising its classifier in the multi-argument event detection phase.

Joint approaches have also been proposed, to avoid the cascading accumulation of errors in pipeline-based approaches, including Markov logic [40], joint inference models [41], search-based models [42], and dual composition-based models [43]. In this latter approach, the dual composition method was applied to solve trigger and argument detection jointly and the resulting system, incorporating the output of the dependency parsing-based event extraction system [39] through stacking, achieved the best performance in the BioNLP-ST’11 GENIA task.

Rule-based approaches have also been attempted [36,44]. The latter system, which detects triggers using dictionaries, and then extracts events by applying rules to syntactic paths, ranked third in the BioNLP’09 ST.

Some event extraction systems have additionally been applied to the entire set of PubMed abstracts [45,46]. The results of event extraction have already been employed in semantic search applications, such as MEDIE [47], UKPMC Evidence Finder [48], applications that perform association mining for knowledge discovery, such as FACTA+ [49] and pathway enrichment and maintenance applications, such as PathText [3,50].

### Meta-knowledge annotation and identification

#### Related work

The automatic assignment of various types of information, fitting into the general definition of meta-knowledge, to different parts of biomedical texts, is already a well-studied problem. The most common types of systems are those that carry out negation/speculation detection [51-57] or perform classification according to rhetorical function (e.g., *BackgroundMethodConclusions*, etc.) [58-64]. Such systems normally operate on continuous text spans, with the sentence being the most common unit of classification. Both rule-based and machine-learning approaches have been attempted, usually using annotated corpora as a starting point.

Concerning the identification of speculation at the sentence level, a number of machine learning approaches have been reported. In [51], an SVM-based text classifier is used to select speculative sentences in abstracts, while in [52,53], weakly supervised machine-learning methods are used to perform simple classification of sentences from full papers on the subject of Drosophila (fruit fly) into speculative or non-speculative. In [53], improvements to the ME classifier were achieved by reducing the feature space size, using both automatic and manual methods and by making use of external dictionaries of hedge clues.

A large amount of work on the detection of negations has focussed on medical reports, for which both rule-based and machine learning approaches have been attempted. In terms of rule-based approaches, [54] presented a system that used regular expressions to find phrases that indicate negation and to filter out sentences containing phrases that falsely appear to indicate negation. The system described in [55] identified negative words or phrases, which were used in conjunction with a parser to identify the scope of the negations. Several machine learning approaches (e.g., [56,57]) learned patterns that indicate negative contexts.

To approach the problem of zone/rhetorical analysis, systems that classify sentences in abstracts (e.g., [59,62,63]) have generally made use of simpler sets of features than systems that operate on full papers. Various learning algorithms have been used for abstract classification, including SVMs [59], linear neural networks [59], Naïve Bayes [62] and conditional random fields (CRF) [63]. All of these approaches use the words (and possibly n-grams of words [63]) in the sentences as features, usually with stemming or lemmatisation. The systems also all consider the position of the sentence within the abstract to be important for accurate classification. In addition, [62] uses feature weighting, while [63] uses contextual features from surrounding sentences. The accurate automatic categorisation of sentences demonstrated in [63] has moreover been integrated with the MEDIE intelligent search system [47].

Similar types of classifiers are used to determine the categories of sentences or zones in full papers, e.g., Naïve Bayes [58,61], SVMs [61], k-nearest nearest neighbour and bigram model [60] and SVMs and CRFs [64]. Some features used are similar to those used in the classification of abstracts, such as words in the sentence, position of the sentence in the abstract and contextual features. However, it is usual that a more complex set of features is employed for full papers. All the systems use syntactic features, while [58] uses several types of semantic features, including the semantic classes of predicates and agents, together with the presence of citations. In [60], document structure features are used, based on the the DocBook standard [65], while [64] employs verb clusters, prediction history and the presence of citations.

Although sentences constitute straightforward and easily identifiable units of text on which to perform annotation and classification, there are a number of reasons why they may be considered too granular to accurately encode the different types of knowledge that are expressed in biomedical text: 

· Expressions of speculation and negations may not apply to the complete sentence

· In terms of rhetorical function / general information content, there may be several types of information expressed in the same sentence

Regarding rhetorical function/general information content, consider the following sentence: 

· *Inhibition of the MAP kinase cascade with PD98059, a specific inhibitor of MAPK kinase 1, may prevent the rapid expression of the alpha2 integrin subunit.*

This sentence contains at least three distinct “nuggets” of information (which are somewhat similar to nano-publication segments), as follows: 

1. A description of an experimental method: *Inhibition of the MAP kinase cascade with PD98059.*

2. A general fact: *PD98059 is a specific inhibitor of MAPK kinase 1.*

3. A speculative analysis: *Inhibition of the MAP kinase may prevent the expression of the alpha2 integrin subunit.*

Thus, systems that are trained to allow only a single classification label per sentence can result in the loss of important information. This problem is partly addressed in [66,67], which report on annotation performed at a finer-grained level, i.e., fragments, of which there may be several in a sentence. Whilst the annotation proposed in [67] uses similar categories to those used for sentence-based rhetorical analysis, the annotation scheme described in [66] is somewhat different, in that it is rather biologically oriented, and identifies 5 different types of information for each fragment (Focus, Polarity, Certainty, Evidence, Direction/Trend). Subsequent work [14] reported on building classifiers based on the annotation introduced in [66] using SVM and ME, achieving a performance in the range of 64–97% F-scores, according to the annotation dimension. The features employed by their classifiers consist of words, bigrams, trigrams, and syntactic chunks, the latter of which were found to be important for the correct assignment of values in the Polarity and Trend (i.e., increase/decrease of a phenomenon or activity) dimensions. The feature space is reduced through the use of several types of processing of the input text, such as stemming and stopword removal. However, the removal of too much information was found to be detrimental to the recognition of some dimensions. For example, tense marking of verbs (which would be removed by stemming) was found to be important for correct classification along some dimensions. It was also found that certain words that are normally classed as stopwords were useful for classification along certain dimensions.

BioScope [68] is a further corpus in which annotations generally consist of smaller units than complete sentences. The corpus annotates the linguistic scopes of negative and speculative keywords, which provides the means to train systems that can determine not only which sentences contain negations or speculations, but also the exact parts of those sentences that have the negative or speculative meaning. The release of this corpus, together with its use in the CoNLL-2010 shared task (which consisted of 2 tasks, i.e., firstly, the detection of speculative keywords and secondly, their scopes) [69], have helped to further stimulate research into negation and hedge detection systems. For example, [70] developed a CRF-based classifier to detect the scope of negations, which was trained on the BioScope corpus, and used words and parts of speech as features. The system was subsequently applied to 336 million sentences to create a database of negated sentences (BioNOT) [71].

Regarding the systems participating in the CoNLL-2010 shared task, a purely rule-based approach was used in [72], in which lexical and syntactic patterns on constituent parse trees and syntactic dependency relations were used in conjunction with a dictionary of known hedge cues. Hybrid approaches were used in [73,74], in which machine learning classifiers were trained to detect hedge cues, using a combination of syntactic and surface oriented features, followed by the application of sets of hand-crafted rules. In [74], the rules were further refined by a CRF classifier. In [75,76], machine learning techniques were applied to both hedge and scope detection, using various combinations of dictionary-based, morphological and syntactic features.

Moving from continuous text spans to event structures, we consider some of the systems that participated in Task 3 of the BioNLP’09 ST, which concerned the detection of negated and speculated events. Several of the features used are the same as those employed by systems operating on continuous text spans, such as syntactic and token features, and dictionaries of negation and speculation clues. The approach described in [44,77] adapted existing modules [78] developed using a different corpus [52] that aim to find dependency relations between dictionaries of negation/speculation clues and event triggers. In [79], a model is trained on BioScope, with additional classification used to discriminate between ambiguous negation/speculation cues, according to context. The system determined whether events were negated/speculated according to whether event triggers and/or their participants fell within the linguistic scopes of the cues. This method was unsuccessful since, as described below, their appears be an incompatibility between linguistically-motivated scopes and biologically-motivated events. In [37], the detection of negation and speculation is carried out using a similar model to the one used for event trigger detection, with the addition of a dictionary of speculation-related words. The types of features used include token features, sentence features, dependency chains and dependency path n-grams.

#### Multi-dimensional meta-knowledge annotation at the event level

While meta-knowledge annotation and identification at the event level has been partially addressed by the GENIA event corpus and the two BioNLP STs, a careful examination of the textual contexts of events reveals that a much richer range of meta-knowledge can often be readily identified, in addition to the basic positive/negative and speculative/non-speculative distinctions. To illustrate this, consider sentences (iii)-(viii) below, all of which contain instances of the same biomedical event organised around different forms of the verb *activate*, as follows: 

· Type: Positive regulation, Trigger: *activate*, Theme: *nitrate reductase operon*, Cause: *narL gene product*

(iii) *It is***known***that the narL gene product activates the nitrate reductase operon*

(iv) *We***examined***whether the narL gene product activates the nitrate reductase operon*

(v) *The narL gene product did***not** *activate the nitrate reductase operon*

(vi) *These results***suggest***that the narL gene product* **might** *be activated by the nitrate reductase operon*

(vii) *The narL gene product* **partially** *activated the nitrate reductase operon*

(viii) **Previous studies***have shown that the narL gene product activates the nitrate reductase operon*

An event extraction system should ideally extract the same event structure as a result of analysing all of the above sentences. However, the interpretation of the event is different in each sentence, according to the sentential context. The expressions in bold represent the clues that lead to these different interpretations. In sentence (iii), the word *known* shows that the event is a generally accepted fact, while the word *examined* in sentence (iv) shows that the event is under investigation and hence that the truth value of the event is as yet unknown. In sentence (v), the word *not* negates the event, meaning that it did not happen, and in sentence (vi), the words *suggest* and *might* show that there is speculation surrounding the event. In sentence (vii), the word *partially* shows that the positive regulation took place with a lesser intensity than would be expected by default, while in sentence (viii), *Previous studies* shows the event refers to previously published knowledge, and hence it is not describing novel information.

Most of the different types of meta-knowledge identified for the events in sentences (iii)–(viii) (with the exception of the partial intensity identified in sentence (vii)) are comparable, at least to some extent, to the meta-knowledge types identified by the systems described in the previous subsection. It may therefore be assumed that meta-knowledge relevant to event interpretation could be “inherited” from one or more of the existing systems that produce sentence, segment or scope-based meta-knowledge annotation. However, such a solution would be problematic for a number of reasons. Firstly, although sentences (iii)–(viii) are very simple sentences containing one event, this will rarely be the case. Similarly to the fact that sentences often contain multiple nuggets of information (which was the argument for the segment-based scheme introduced in [66]), most sentences will contain multiple events, each of which could have a different meta-knowledge interpretation. Thus, inheriting event meta-knowledge from sentence-based classifications would not be appropriate in most cases.

In terms of the feasibility of inheriting meta-knowledge from smaller spans of text, it may sometimes be the case that a particular textual fragment or negation/speculation scope corresponds approximately to the span of text from which an event trigger and its arguments are drawn. However, in other cases, this will not be so; in contrast to scopes and segments, events do not constitute continuous spans of text, meaning that event triggers and participants may be drawn from multiple fragments of a sentence. This means that, even though the meta-knowledge dimensions identified by the classifiers described in [14] are very similar to the different types of meta-knowledge that can be identified for the events in sentences (iii)–(viii), the lack of a straightforward mapping between textual segments and events makes it difficult to use such classifiers to facilitate the interpretation of events. As was mentioned above, a similar lack of compatibility can be observed between linguistically-motivated negation and speculation scopes, and biologically-motivated events that fall (partially) within these scopes. An analysis of the differences between the scope-based annotation in BioScope and events annotated as negated or speculated in the original GENIA event annotation showed that only 51% of events in the GENIA event corpus with event triggers occurring within a speculated/negated scope were actually annotated as speculated/negated themselves [80].

Thus, in terms of the automatic assignment of meta-knowledge at the level of events, it is clear that separate event-based classifiers must be constructed. The feasibility of training such classifiers can be greatly increased through the use of a corpus in which meta-knowledge also been assigned at the event level. Accordingly, our EventMine-MK system is trained using such a corpus, i.e., the GENIA-MK corpus, which includes detailed event-level meta-knowledge information. Below, we provide brief details of this corpus and the scheme used to annotate it.

#### Event-based meta-knowledge annotation scheme

The meta-knowledge enrichment of the GENIA event corpus, which is carried out in strict accordance with a formally-defined annotation scheme [20], consists of the classification of each event along five different meta-knowledge dimensions, as well as the annotation of clue expressions for each dimension (such as those emboldened in the example sentences (iii)–(viii)), whenever such expressions are present in the sentence. The annotation scheme used to create the GENIA-MK corpus thus aims to provide a richer representation of meta-knowledge than is annotated in the GENIA event corpus and the two BioNLP STs. The scheme is very much inspired by the one described in [66] and the encouraging results obtained by a system trained on the resulting corpus [14]. However, our scheme was tailored to the interpretation of bio-events, through the examination of a large number of events and their contexts when designing the scheme. Unlike many of the other annotated corpora introduced above, but in common with the BioScope corpus, we annotate clue words/expressions that are used to determine the specific values assigned to the different meta-knowledge dimensions. Our decision to annotate such clues was motivated by the fact that several of the meta-knowledge assignment systems introduced above, including those participating in the detection of negated and speculated events in the BioNLP’09 ST, have used dictionaries of clue words as features. Thus, it is to be expected that clues will also be important in the assignment of other types of meta-knowledge at the event level. Clues for several of our meta-knowledge dimensions have not previously been annotated in other corpora, nor have clues for negation and speculation at the event level. We thus considered it important to identify a set of event-based clue expressions, which could subsequently be used to compile dictionaries.

**Figure 2:**
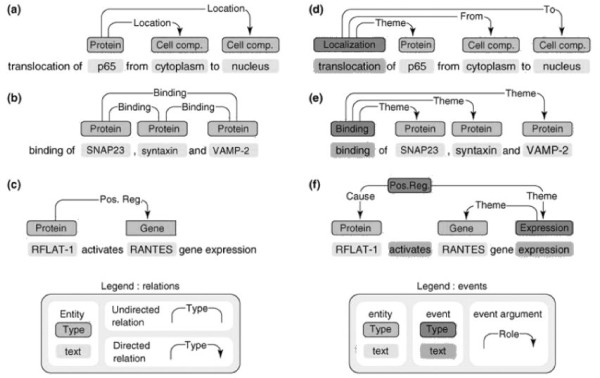
Meta-knowledge annotation scheme.^*^ denotes the default value for each dimension.

Figure 2 provides an overview of the meta-knowledge annotation scheme. The boxes with the light grey (dotted) background correspond to information that is common to most bio-event annotation schemes (the participants in the event, together with an indication of the class or type of the event). The boxes with the dark grey (striped) backgrounds correspond to meta-knowledge annotation dimensions and their possible values, whilst the white box shows the hyper-dimensions that can be derived by considering a combination of the annotated dimensions. High levels of inter-annotator agreement were achieved [10] between the two annotators involved (a biologist and a linguist), falling in the range of 0.84–0.93 (Cohen’s kappa [81]).

The five dimensions of meta-knowledge that make up the scheme are described in more detail below: A default value is specified for each dimension, which is assigned if there is no explicit evidence for the assignment of one of the other values. 

· **Knowledge Type (KT)** refers to the general information content of the event. Possible values are *Investigation* (enquiries or investigations), *Observation* (direct observations), *Analysis* (inferences, interpretations, speculations or other types of cognitive analysis), *Fact* (general facts and well-established knowledge), *Method* (experimental methods), and *Other* (default value; assigned to events that either do not fit into one of the above values, do not express complete information, or whose KT is unclear or is assignable from the context).

· **Certainty Level (CL)** identifies events for which there is explicit indication that there is less than complete confidence in the event. There are 3 possible values: *L1* (low confidence or considerable speculation, or events which occur infrequently), *L2* (high, but not complete, confidence, or events that occur frequently, but not all of the time) and *L3* (default value; no explicit mention of either uncertainty or that the event does not occur all of the time).

· **Polarity** encodes the truth value of the event, with two possible values, i.e., *Positive* (default value) and *Negative*. A negated event is defined as one that describes the absence or non-existence of an entity or a process.

· **Manner** refers to the rate, level, strength or intensity of the event, with three possible values, i.e., *High*, *Low* and *Neutral* (default value).

· **Source** determines the origin of the knowledge expressed by the event (i.e., the current paper or a previous study), with its values *Current* (default value) and *Other*.

**Hyper-dimensions** correspond to additional information that can be inferred by considering combinations of some of the explicitly annotated dimensions. We have identified two such hyper-dimensions, each with binary values (*Yes* or *No*): **New Knowledge** (inferred from KT, Source and CL) and **Hypothesis** (inferred from KT and CL).

The following two sentences serve to exemplify meta-knowledge annotation of events: 

· *Each of these domains possessed strong homology to motifs previously found to bind the cellular factor NF-kappa B.* (PMID: 2148290 [82])

Sentence (ix) contains the following event. 

· Type: Binding, Trigger: *bind*, Theme: *motifs*, Theme: *NF-kappa B*

Here, *Binding* is a multi-participant binding event, and it takes multiple Theme arguments. The meta-knowledge annotation for the event is as follows: 

· KT: Observation, clue: *found*

· CL: L3

· Polarity: Positive

· Manner: Neutral

· Source: Other, clue: *previously*

No clue expressions are annotated for CL, Polarity and Manner, and they retain their default values, since there is no evidence in the sentence that refers explicitly to these meta-knowledge annotation dimensions. 

· *This Delta19 beta-catenin mutant localizes to the nucleus because it may not be efficiently sequestered in the cytoplasm.* (PMID: 10330189 [83])

Sentence (x) contains the following event, among others. 

· Type: Localization, Trigger: *sequestered*, Theme: *Delta19*

The meta-knowledge annotation for the event is as follows: 

· KT: Analysis, clue: *may*

· CL: L1, clue: *may*

· Polarity: Negation, clue: *not*

· Manner: Neutral

· Source: Current

In this example, the same clue expression, *may*, is used for both the KT and CL dimensions. The word *may* primarily denotes high speculation. However, speculation inherently involves cognitive analysis, and since there is no other *Analysis* clue expression present in the sentence, *may* is also annotated as the KT clue.

The assignment of meta-knowledge at the event level can be seen as complementary to zoning/rhetorical or augmentation analysis methods, in that it can provide a finer-grained analysis of the various types of information that can occur within a particular text zone. The methods operating at different levels of text granularity could be used simultaneously in text mining systems, e.g., the identification of particular coarse-grained textual regions could be used as an initial filter for locating events with particular types of meta-knowledge. For instance, *Conclusion* sentences would normally be expected to contain events that describe fairly definite analyses of events.

## Methods

In this section, we begin by providing a brief overview of EventMine [19], i.e., the existing event extraction system that forms the basis of the integrated EventMine-MK system. Subsequently, we describe the two stages of our work in creating our new integrated system, which can not only extract events but also assign meta-knowledge to them. Firstly, we present our meta-knowledge assignment system, which attaches meta-knowledge information to pre-recognised events. We explain how the performance of this system was intrinsically evaluated by applying it to manually annotated events in the GENIA-MK corpus. Secondly, we provide an account of how the meta-knowledge assignment system was integrated with the existing EventMine system, to create the new EventMine-MK system. Finally, we describe the EventMine-MK UIMA/U-Compare component.

### Event extraction system: EventMine

**Figure 3:**
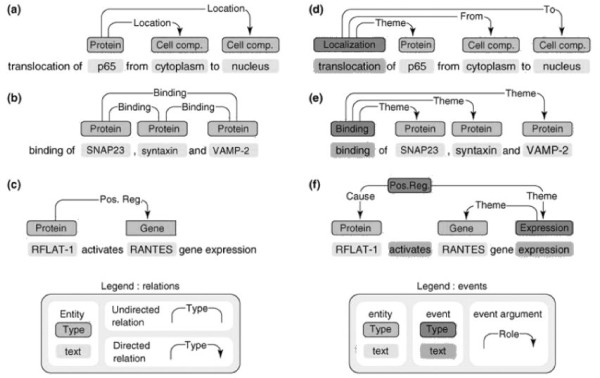
**EventMine event extraction pipeline.** The diagram illustrates the pipeline model used by EventMine. The system takes as input texts in which proteins/genes have already been identified. Trigger/entity detection classifies appropriate words in each sentence as triggers or entities, argument detection finds semantic pair-wise relations among event participants and multi-argument event detection merges several relations into events. This sentence is taken from PMID: 9341193 [85].

In this section, we provide an overview of the existing EventMine [19] system, and describe some modifications that have been made as part of the development of the EventMine-MK system. When applied to the ST corpus [24], EventMine outperformed other systems participating in the BioNLP’09 ST subtask Task 2 (i.e., the identification of secondary arguments, such as location and site). EventMine is a machine learning-based pipeline system with three detection modules: event trigger/entity detection, event argument detection and multi-argument event detection. Trigger/entity detection assigns an appropriate trigger/entity category (e.g., *BindingEntity*) to each word that potentially constitutes the head word of an event participant; argument detection finds semantic pair-wise relations among event participants; and multi-argument event detection merges several relations into events. Figure 3 illustrates the flow of event extraction. In each module, EventMine solves multi-class multi-label classification problems using L2-regularised L2-loss SVM (L2-SVM) with a one-vs-rest classification scheme [84]. That is, binary classifiers are built which distinguish one of the labels from the rest. Classification of new instances using the scheme for the multi-class multi-label classification is carried out by assigning to the instances both the labels suggested by the classifiers and a label with the highest classification score, the latter of which is employed to ensure that all instances are assigned a label.

**Figure 4:**
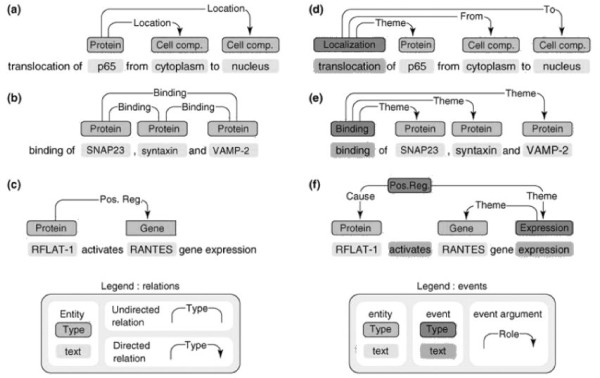
**Shortest dependency path example.** This example illustrates the shortest dependency path between *IEXC29S* and *transactivate* on the dependency tree produced by the GDep beta2 parser [87] (SUB: Subject, OBJ: Object, AMOD: Modifier of Adjective or Adverbal, VMOD: Modifier of Verb, PRD: Predicative Complement). The shortest path is shown in bold.

EventMine is designed to extract event structures from parser output. Any dependency parser could be substituted. However, in the system described in this paper, we have used a combination of the Enju parser [86] and the GDep parser [87], following [24]. In EventMine, five base functions have already been implemented and provided to extract features representing a word or a pair of words, together with their immediate textual contexts in the sentence. These functions are as follows: 

1. *Token feature function* – extracts the surface representation of a word. The features extracted consist of character types (e.g., number, symbol), character n-grams (n=1; 2; 3; 4) (e.g., *t*, *r*, ⋯, *tr*, *ra*, ⋯, *tra*, *ran*, ⋯, *tran*, *rans*, ⋯ for *transactivate*), base form of the word (e.g., *be* for *were*) and part-of-speech (POS) of the word (e.g., *VBD* for *were*).

2. *Neighbouring word feature function* – extracts all 2-step dependency paths from the target word, which then are used to extract n-grams. For example, one of the 2-step paths is *were* ←*PRD*–*unable* ← *AMOD*–*transactivate* for *transactivate* in Figure 4. The features used consist of: the features extracted by the token feature function for each word, word and dependency n-grams (n=2; 3; 4) (e.g., *be* ← *PRD*–, ←*PRD*–*unable* ←*AMOD*–), word n-grams (n=2; 3) (e.g., *be unable*, *unable transactivate*, *be unable transactivate*) and dependency n-grams (n=2) (e.g., ←*PRD*– ←*AMOD*–). In the n-grams, each word is represented by its base form.

3. *Word n-gram feature function* – extracts word n-grams (n=1; 2; 3; 4) within a window of three words before or three words after the target word. Each word is represented by its base form, POS and its relative position (before or after the target word). For example, the function extracts word n-grams from *unable to significantly transactivate the c-sis/PDGF-B* for *transactivate* in Figure 4.

4. *Pair n-gram feature function* – extracts word n-grams (n=1; 2; 3; 4) within a window of three words before the first word in the target pair and three words after the last word. Each word is represented by its base form, POS and its relative position (before, between or after the target pair). For example, for the trigger/argument pair *transactivate* and *c-sis/PDGF-B* in Figure 4, the window from which features are extracted is *unable to significantly transactivate the c-sis/PDGF-B promoter*. The word sequence *unable to significantly* is before the pair, *transactivate the c-sis/PDGF-B* is between the pair, and *promoter* is after the pair.

5. *Shortest path feature function* – extracts the shortest dependency paths (e.g., [88]) between a word pair. Figure 4 illustrates the shortest path between *IEXC29S* and *transactivate*. Several types of information are extracted to represent the shortest paths, including their length (e.g., 3), word n-grams (n=2; 3; 4), dependency n-grams (n=2; 3; 4), consecutive word n-grams (n=1; 2; 3) representing governor-dependent relationships (e.g., *IEXC29S* →*bebe* ←*unable*), edge walks (word-dependency-word) and their sub-structures (e.g., *IEXC29SSUB* → *beIEXC29SSUB* →), and vertex walks (dependency-word-dependency) and their sub-structures (e.g., –*SUB*→ *be* ←*unable*–, –*SUB* → ←*unable*–). Each word is represented by its base form.

The three modules in EventMine are implemented by using different combinations of these functions, details of which are provided in [19].

As part of the construction of the EventMine-MK system, we have incorporated two modifications into original EventMine system, in an attempt to improve the performance of the event extraction functionality. Firstly, a dictionary matching-based filter was incorporated into the trigger/entity detection to reduce calculation time, by ensuring that only words that match dictionary entries are considered as trigger/entity candidates, instead of all words in the text. The dictionary was created by firstly extracting trigger and entity head words from the training data, and then expanding this initial list by using two different resources to improve coverage. From the UMLS SPECIALIST lexicon [89], we obtained synonyms of words in the original list, as well as derivationally related words (e.g., phosphorylate→ phosphorylation). Using WordNet 3.0 [90], we added words that were linked to those in the initial list via the *hypernyms* and *similar to* relations.

Secondly, similarly to [91], we used feature hashing to reduce memory usage. Feature hashing maps features into a fixed space (we used 2^20^ features), and does not require string to integer mapping to maintain feature indices.

These modifications caused a very small decrease in the performance of the system on the BioNLP’09 ST subtask Task 2 (around 0.1% F-score). However, they almost halved the computation time and reduced the memory usage by approximately 75%.

### Meta-knowledge assignment system

As a starting point for the development of EventMine-MK, we firstly constructed a system that assigns meta-knowledge to pre-annotated events in the GENIA event corpus, through training on the GENIA-MK corpus. This system thus allows an intrinsic evaluation of the meta-knowledge assignment task to be carried out, independently of the event extraction task. The meta-knowledge assignment problem is treated as a multi-class classification problem for each dimension, i.e., KT, CL, Polarity, Manner and Source, since each event is always assigned a single value for each of the five dimensions. Although the aim of the system described in [14] is somewhat similar, in that multiple meta-knowledge values are assigned to sentence fragments, that system used a multi-class, multi-label classification approach, since their annotation permitted multiple labels to be assigned to the same text fragments for a given annotation dimension.

In order to perform classification in our meta-knowledge assignment system, features are extracted from the target event, as explained below, and they are fed to an SVM classifier. In the same way as in the event extraction pipeline, the type of classifier used is L2-SVM with a one-vs-rest classification scheme. Two classification configurations are employed. Firstly, *biased regularisation factors*[84] are introduced for positive examples, both to alleviate the problem in the one-vs-rest classification scheme that negative examples constitute a sizable proportion of the training data examples, and also to improve the stability in predicting infrequent meta-knowledge values. Regularisation factors for positive examples are assigned by calculating the ratio of negative to positive examples for each class. The regularisation factor for negative examples is set to 1. Secondly, *type-based feature normalisation* is also employed to reduce the effects of the different feature scales: the features in each type are normalised using the L^2^ norm (Euclidean length) to form a unit vector [92], and the whole feature vector is then normalised using the L^2^ norm.

With regard to features, we employed two types: event structure-based features and features for specific meta-knowledge values.

Since we are dealing with meta-knowledge annotated at the event level, some of the features should reflect event structures. Accordingly, some of the features we use are comparable to those used by the systems trained to extract negated and speculated events from the BioNLP ST corpora, e.g., [37,44,77]. We have used the same extraction functions employed by EventMine to extract the following three types of features: 

1. *Meta-knowledge clue features* – represent the shortest dependency paths between event participants (trigger and arguments) and meta-knowledge clue expressions. The features are extracted using the *shortest path feature function*. Here, in common with several other approaches, clue expressions are extracted by matching with clue word lists. The clue word lists are constructed by selecting the most suitable clue words from all the clue words in the BioScope corpus and the training part of the GENIA-MK corpus. The selection is performed using pointwise mutual information (PMI) [93], which measures the level of association between a clue word and its meta-knowledge values. The threshold for PMI was set at -1.5 in order to minimise the number of ambiguous clue words that are extracted. The exact value of the threshold was determined manually, based on the results of 10-fold cross validation on the training data.

2. *Trigger features* – represent the contexts around the event trigger, extracted using the *neighbouring word feature function*.

3. *Trigger-argument pair features* – extracted using the *pair n-gram feature function*.

A manual analysis of the meta-knowledge annotation in the GENIA-MK corpus revealed that the presence of specific types of clue words and phrases is not the only important factor in determining the correct meta-knowledge values to assign for certain dimensions. Based on this analysis, together with an examination of features used in related systems, we added an additional set of features that attempt to capture other characteristics of the text that are important in determining the correct meta-knowledge values to assign. The additional features can be split into two types. 

1. *Sentence features* refer to both the absolute position (e.g., second sentence, third sentence) and the relative position (e.g., 0.25 (= 2/8) for the second of eight sentences) in the abstract of the sentence that includes the event trigger. The features are used according to the observation that certain types of meta-knowledge (particularly events belonging to different values within the KT dimension) tend to appear in fixed places in abstracts (e.g., events with the KT type *Fact* or *Observation* often appear towards the beginning of an abstract). Similar features are used in nearly all systems that assign rhetorical functions or sentence categories, e.g., [58,59,61-64], some of whose values are comparable to those in our KT dimension.

2. A *citation feature* refers to the existence of citations. Citations are extracted via a regular expression that matches parentheses or brackets surrounding numbers (e.g., *[108]*) or sequences ending in 4 digits (e.g., (..., 1998)). Clues for *Other* (Source dimension) often constitute citations, and thus are not covered by the clue dictionaries. Such features have also been used in other systems that aim to identify parts of the text where other work is referred to, e.g., [58,61,64].

Our system has two potential limitations in tackling the problem, as follows: Firstly, the system takes into account neither dependencies between meta-knowledge values in different events, nor dependencies among the different dimensions of the same event. However, we will show that even by ignoring such dependencies, our system performs well for most meta-knowledge dimension values. Secondly, the current version of our system is also not able to extract specific associations between meta-knowledge dimension values (e.g., *Observation*) and their clue expressions (e.g., *found*). The ability to extract some associations may, however, be useful in certain situations, e.g., to present meta-knowledge instances to users with textual evidence highlighted. Accounting for these dependencies and associations is left as future work.

### Integrating the meta-knowledge assignment system with EventMine

**Figure 5:**
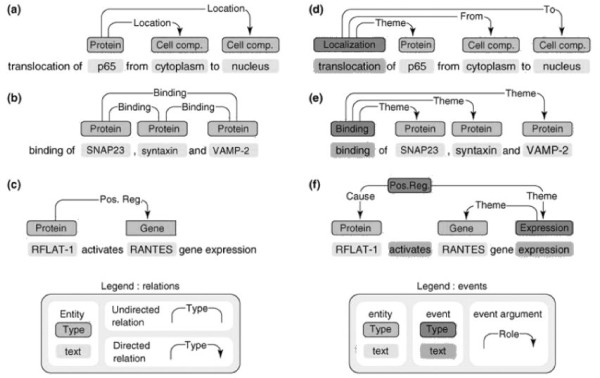
**EventMine-MK event extraction pipeline.** The diagram illustrates the pipeline model used by EventMine-MK. The meta-knowledge assignment module is applied after event extraction. The functionality of the trigger/entity detector is different from the one shown in Figure 3, in that meta-knowledge clues are now additionally detected by this module. The extracted clues are used as features by the newly-added meta-knowledge extraction module.

In order to perform the complete task of fully automatic extraction of both events and their associated meta-knowledge information, the meta-knowledge assignment system described in the previous section has been integrated with EventMine, as a module in the event extraction pipeline that is executed following the detection of multi-argument events. The meta-knowledge clues are recognised by treating them as new classes of words to be identified and classified by the trigger/entity module. Based on these recognised meta-knowledge clues, features are extracted for use by the meta-knowledge assignment system. Figure 5 illustrates the updated flow of extraction.

In order to train the EventMine-MK system, we transferred the meta-knowledge annotation of the GENIA-MK corpus to the training and development portion of the ST corpus (henceforth referred to as “the ST-MK corpus”), whose events consist largely of a modified subset of the events that are present in the GENIA event corpus. Given the current state-of-the-art in event extraction, using the complete GENIA-MK corpus for training would not allow a practical system to be produced, for a number of reasons: 

· The GENIA event corpus is annotated with nested, fine-grained NEs, which belong to 47 different classes. As an example of this nesting, the string *HIV-1 gene* is annotated in the GENIA event corpus as an entity of type *DNA domain or region*. However, the nested string *HIV-1* has additionally been identified as an entity of type *Virus*. Although there exist several systems that can extract nested NEs, this is mostly done using a more coarse-grained set of NE classes (e.g., [94]).

· The extraction of fine-grained nested NEs has been attempted [95], which achieved a micro F-score performance of 67.3%, using fragment matching. The recognition performance amongst the different classes ranged from 7.4% to 80.7%, which is considerably lower than coarse-grained NE extractors.

· Extraction of fine-grained events. When applied to the GENIA event corpus (36 classes of events), the best performance achieved by EventMine was 34% F-score, given the fine-grained NEs annotations. In comparison, the EventMine system achieved a performance of greater than 55% F-score on the ST corpus.

It was possible to transfer meta-knowledge annotations from the GENIA-MK corpus to over 90% of events in the ST corpus (9,494/10,410 events). The remaining events in the ST corpus correspond to new annotations added when the corpus was created, which were not based on events in the GENIA event corpus. In order to prevent errors in the transfer of meta-knowledge between the two corpora, the annotation from the GENIA-MK corpus was transferred to the ST corpus only when events in the two corpora could be matched in terms of their event types, event IDs and core argument text spans. The event IDs were provided by the ST organisers, since the event IDs in the ST corpus are different from the event IDs in the GENIA event corpus. In order to verify the correctness of the transfer process, we randomly examined 100 events in the two corpora, and found that meta-knowledge had been correctly transferred in all cases. The distribution of event instances in the two corpora, in terms of the meta-knowledge values assigned, is shown in Table 2.

**Table 2:** Distribution of instances by frequency and percentage (%) for each meta-knowledge value

**Value**	**GENIA**		**ST**		**ST(%)/GENIA(%)**
	**frequency**	**%**	**frequency**	**%**	
Investigation	1,914	5.3	520	5.5	1.03
Analysis	6,412	17.8	1,448	15.3	0.86
Observation	12,479	34.7	3,892	41.0	1.18
Fact	2,900	8.1	570	6.0	0.75
Method	973	2.7	40	0.4	0.16
Other	11,309	31.4	3,024	31.9	1.01
L3	33,090	91.9	8,921	94.0	1.02
L2	2,148	6.0	485	5.1	0.86
L1	749	2.1	88	0.9	0.45
Positive	33,779	93.9	8,939	94.2	1.00
Negative	2,208	6.1	555	5.8	0.95
High	1,381	3.8	466	4.9	1.28
Low	322	0.9	112	1.2	1.32
Neutral	34,284	95.3	8,916	93.9	0.99
Current	35,447	98.5	9,370	98.7	1.00
Other	540	1.5	124	1.3	0.87
Event Total	35,987	100.0	9,494	100.0	1.00

The last column shows the ratio of the percentage of events assigned the indicated value in the ST corpus to the percentage in the GENIA-MK corpus. Events that had no triggers were ignored.

In contrast to the GENIA event corpus, the ST corpus contains only 9 event types and 1 NE category. Moreover, the ST corpus only contains around one third of the events present in the GENIA event corpus. Hence, two thirds of the meta-knowledge annotations in the GENIA-MK corpus cannot be transferred to the ST-MK corpus. In order for useful information contained in these unused annotations from the GENIA-MK corpus not to be lost in the model trained on the ST-MK corpus, the output of the meta-knowledge assignment model that was trained on the complete GENIA-MK corpus is used to create additional features that are used in the assignment of meta-knowledge by EventMine-MK. These additional features include prediction scores that are made by the GENIA-MK-trained meta-knowledge assignment model for all meta-knowledge values.

### Integration into U-Compare/UIMA

U-Compare [22] is an integrated text mining (TM) / natural language processing (NLP) system based on the Unstructured Information Management Architecture (UIMA) framework. U-Compare provides a large number of UIMA-compatible TM/NLP components, including sentence splitters, NE recognisers, parsers, etc. Its graphical user interface makes it easy for users to define, run and visualise the results of workflows, including the ability to compare the results of using different components for the same task. We constructed a UIMA component for our EventMine-MK system (also available as a UIMA-compatible web service), which takes as input a sentence with NE annotation and outputs events contained within the sentence, with meta-knowledge information assigned. The component can be inserted into any UIMA-based workflow (built using the U-Compare workflow interface or other means), provided that previous components in the workflow have split the input document into sentences and identified appropriate NEs. The EventMine-MK component is available as part of the current U-Compare software download [96]. The component is named “EventMine MetaKnowledge” and is classified under “Event detectors” within the library of U-Compare components.

## Results and discussion

### Evaluation settings

The corpora (the GENIA-MK event corpus and the ST corpus) were split into sentences using the GENIA sentence splitter [97], and the resulting sentences were processed using two parsers, i.e., the Enju 2.4.1 parser with the GENIA model [86] and the GDep beta2 parser [87]. EventMine-MK employed parse results produced by both parsers, as in the original EventMine system [19]. EventMine follows most other event extraction systems in employing syntactic parsers with biomedical models [9,23]; the impact of such parsers on event extraction has been analysed in [24,98].

Liblinear-java 1.7 [84] was employed as the classification tool used to perform all types of classification in EventMine-MK (relating to both event extraction and meta-knowledge assignment), with the bias term set and the regularisation value C set to 1 for negative examples.

For the purposes of training and testing the meta-knowledge assignment system, the GENIA-MK corpus was partitioned into a training set (800 abstracts) and a test set (200 abstracts). The training set contains the same abstracts as the ST training set, while 150 of the abstracts in the GENIA test set were the same as those contained in the development set of the ST. Each event was represented by an event trigger and core arguments (*Theme* and *Cause*). The other arguments were ignored to simplify the problem, since additional arguments relating to locations, experimental conditions, etc., rarely have an impact on the meta-knowledge values that are assigned to events. If arguments consisted of multiple discontinuous spans, such spans were replaced with the smallest continuous span that covered the discontinuous spans. The representation of events assumed by our system includes event triggers, in order to extract certain types of features. Accordingly, the small number of events in the GENIA-MK corpus that were not annotated with explicit triggers was ignored (i.e., 673 out of 29,025 events in the training set and 198 out of 7,833 events in the test set). In terms of the ST corpus used to develop EventMine-MK, the training and development portions used in the ST were employed as the training and test sets, respectively.

Evaluation of meta-knowledge assignment was performed based on precision, recall, F-score and their macro-averages or micro-averages for each category. The assignment of the majority value for each annotation dimension (Majority) was employed as our baseline.

Evaluation of negated and speculated event extraction (Task 3) on the ST corpus was carried out according to the ST settings. 800, 150 and 260 abstracts were used as the training, development and test sets, respectively. The ST evaluation server was used to perform the evaluation, and the results are reported according to Approximate Span Matching/Approximate Recursive Matching evaluation criteria [9]. The evaluation was performed by matching gold standard events and their negation/speculation annotations, so that both event extraction performance and negation/speculation detection performance are taken into account. Precision, recall and F-score, together with their micro-averages, are used to report the results.

An analysis was carried out of the differences between meta-knowledge distribution in abstracts and full texts, using the ST-MK corpus, which consists of abstracts, and the full-text subset of BioNLP-ST’11 GENIA corpus. The training and development sets in the ST-MK corpus were used for training purposes, while the predictions on the ST corpus test set and the full-text subset were used to perform the analysis. The BioNLP-ST’11 GENIA evaluation server was used to perform the evaluation of negated and speculated event extraction on the full texts.

### Meta-knowledge assignment to GENIA events

In order to construct the meta-knowledge assignment system, we firstly used 10-fold cross validation to evaluate the system using the training set of the GENIA-MK corpus, using all the features described in the *Meta-knowledge assignment system* section of *Methods*, together with the use of both configuration settings, i.e., biased regularisation factors and type-based feature normalisation. Subsequently, in order to evaluate the contribution of different features and settings, we trained various other versions of the system, by disabling one feature or configuration type at a time. The evaluation results obtained for all versions of the system are summarised in Table 3.

**Table 3:** Meta-knowledge assignment results using 10-fold cross validation on the GENIA-MK training set with various settings

**Meta-knowledge**		**Disabled features**					**Disabled learning settings**			
		**-Meta-**	**-Trigger**	**-Trigger-**	**-Sentence**	**-Citation**	**-Type-based**	**-Biased**	
**Dimension**	**Value**	**knowledge**	**features**	**argument**	**features**	**features**	**feature**	**regularisation**	**ALL**	**Majority**
		**clue features**		**pair features**			**normalisation**	**factors**		
KT	Investigation	71.9	61.2	71.7	70.8	71.6	73.2	70.3	71.8	0.0
	Analysis	75.6	68.1	74.6	75.6	75.7	75.7	75.0	75.8	0.0
	Observation	74.7	71.1	72.7	71.7	75.1	74.0	74.6	75.3	41.4
	Fact	67.2	65.5	59.6	56.2	66.8	59.3	64.5	67.3	0.0
	Method	61.9	56.0	52.8	61.6	61.7	55.3	52.9	62.0	0.0
	Other	74.1	71.6	73.5	73.9	74.7	74.0	74.5	74.8	0.0
	Macro	70.9	65.6	67.5	68.3	70.9	68.6	68.6	71.2	6.9
	Accuracy	73.6	69.4	71.6	71.6	73.9	72.7	73.3	74.1	26.1
CL	L3	97.4	97.6	97.7	97.8	97.8	97.8	98.0	97.8	94.5
	L2	67.1	69.7	72.0	72.8	73.0	72.6	70.9	73.0	0.0
	L1	76.4	78.7	76.0	78.1	78.7	75.9	78.7	78.6	0.0
	Macro	80.3	82.0	81.9	82.9	83.2	82.1	82.5	83.1	31.5
	Accuracy	95.0	95.4	95.5	95.8	95.8	95.7	96.2	95.8	89.6
Polarity	Positive	97.7	97.3	97.1	97.6	97.5	97.4	98.1	97.5	95.1
	Negative	65.9	65.0	64.1	67.8	67.3	64.2	63.6	67.3	0.0
	Macro	81.8	81.2	80.6	82.7	82.4	80.8	80.9	82.4	47.5
	Accuracy	95.8	95.0	94.7	95.5	95.4	95.1	96.4	95.4	90.7
Manner	High	44.1	41.3	32.0	43.7	42.3	37.0	10.0	42.6	0.0
	Low	17.6	13.5	14.7	18.4	17.1	13.3	0.8	17.1	0.0
	Neutral	97.3	97.1	95.4	97.1	97.0	96.9	97.6	97.0	95.9
	Macro	53.0	50.6	47.4	53.1	52.1	49.1	36.1	52.2	32.0
	Accuracy	94.7	94.4	91.1	94.3	94.1	93.9	95.4	94.1	92.1
Source	Current	99.1	98.6	98.5	99.1	98.9	99.1	99.3	98.9	98.6
	Other	43.7	32.2	35.4	41.1	40.6	42.8	18.3	41.9	0.0
	Macro	71.4	65.4	66.9	70.1	69.8	70.9	58.8	70.4	49.3
	Accuracy	98.2	97.3	97.0	98.2	97.9	98.2	98.6	97.9	97.2

“-” means that the named feature type or configuration is disabled. The F-score for each meta-knowledge value, together with the macro-averaged F-score and accuracy (= Micro-averaged F-score) for each dimension, are reported. The macro-averaged F-score is calculated by taking the average of the F-scores for each different meta-knowledge value within the dimension.

With regard to the highest macro F-score, the best setting differs, depending on the dimension under consideration. Event structure-related features (Trigger and Trigger-argument pair features) are seen to be crucial for achieving a higher performance, since, for most dimensions, the macro F-score drops considerably when these features are disabled. For other features, their impact was generally relatively small, and our experiments showed that disabling some features could lead to positive as well as negative impacts on the performance of the system.

According to the results reported in Table 3, the use of the meta-knowledge clue features generally has a positive effect on the assignment of KT, CL and Polarity values. However, the results also suggest that these features can have a slightly negative impact on the assignment of Manner and Source values. A possible reason for this is that there are few commonly occurring clues for values of these dimensions. For example, there are only 6 clues for the *Low* value of the Manner dimension that occur 10 or more times in the entire GENIA-MK corpus, compared to 88 clues for the *Analysis* value of the KT dimension that occur 10 or more times. If a stable set of clues cannot be identified in the training set, clues will be rarely matched in the test set, and thus the existence of the clues may confuse the classifier.

Sentence features are useful for KT, CL and Source, but not for Polarity and Manner. This seems reasonable, since there is often some kind of correlation between the position of the sentence in the abstract and the possible values of the KT, CL and Source dimensions. For example, in terms of the KT attribute, events representing facts will often occur towards the beginning of the abstract, while events denoting analyses will often come after results have been reported, i.e., towards the end of the abstract. The same characteristics would thus apply to CL, since this dimension is only applicable to events with a KT value of *Analysis*. For the Source attribute, the non-default value of *Other* is applicable when the event describes work that was not carried out as part of the current study, which is most likely to be mentioned at the beginning of the abstract. The same position-based feature would not normally be applicable to Polarity, since events of any type may be negated. Similarly, expressions of high or low Manner can apply to any event describing a biological process, whether the event describes a fact, observation, analysis, etc. As would be expected, citation features have the most positive impact on the assignment of the *Other* value of the Source dimension. For other dimensions, the effect of this feature is negligible. The highest accuracy is produced when biased regularisation factors are removed, with the exception of the KT dimension. However, the same system setting also produced the lowest macro F-score, by a considerable margin, for the Manner and Source dimensions, since the removal of biased regularisation means that infrequent meta-knowledge values are not predicted well.

Our results also show that it is safe to use all the features and configurations for the prediction of all dimensions, since the difference between the performance when using the best setting, and the performance when using all features and configurations, is less than 1%. According to the results of these experiments, we decided to use the system setting with all the features and configurations enabled for all subsequently reported experiments, unless otherwise stated. We do not use the best setting for each dimension, since calculating and storing a different set of features for each of the five dimensions would require extra computational and spatial costs.

Following this initial set of experiments, we applied the meta-knowledge assignment system to the test set of the GENIA-MK corpus, and the results are summarised in Table 4. The performance on the test set is generally better than the baseline, except for a small degradation in F-score for the *Neutral* value of the Manner dimension and the micro-averaged F-score for the Manner dimension. The performance is somewhat low for rarely appearing values, including *Low*, *High*, *Method* and *Other* (Source). Precision and recall are similar to each other in all cases, except for the *Low* value of the Manner dimension. This shows that, for the most part, biased regularisation factors are able to keep a balance between precision and recall, even for infrequent meta-knowledge values.

**Table 4:** Meta-knowledge assignment results on events in the GENIA-MK test set

**Dimension**	**Value**	**Count**	**R / P / F**	**Majority (R / P / F)**
KT	Investigation	411	69.1 / 75.5 / 72.2	0.0 / 0.0 / 0.0
	Analysis	1,340	70.4 / 78.4 / 74.2	0.0 / 0.0 / 0.0
	Observation	2,593	72.1 / 72.3 / 72.2	100.0 / 34.0 / 50.7
	Fact	673	63.7 / 68.0 / 65.8	0.0 / 0.0 / 0.0
	Method	178	53.4 / 54.3 / 53.8	0.0 / 0.0 / 0.0
	Other	2,440	77.0 / 70.6 / 73.6	0.0 / 0.0 / 0.0
	Macro Average	7,635	67.6 / 70.0 / 68.6	16.7 / 5.7 / 8.5
	Micro Average	7,635	72.0 / 72.0 / 72.0	34.0 / 34.0 / 34.0
CL	L3	7,057	97.6 / 97.5 / 97.6	100 / 92.4 / 96.1
	L2	420	68.6 / 64.6 / 66.5	0.0 / 0.0 / 0.0
	L1	158	67.1 / 84.8 / 74.9	0.0 / 0.0 / 0.0
	Macro Average	7,635	77.8 / 82.3 / 79.7	33.3 / 30.8 / 32.0
	Micro Average	7,635	95.4 / 95.4 / 95.4	92.4 / 92.4 / 92.4
Polarity	Positive	7,201	97.3 / 98.0 / 97.7	100 / 94.3 / 97.1
	Negative	434	67.1 / 60.1 / 63.4	0.0 / 0.0 / 0.0
	Macro Average	7,635	77.8 / 82.3 / 79.7	50.0 / 47.2 / 48.5
	Micro Average	7,635	95.6 / 95.6 / 95.6	94.3 / 94.3 / 94.3
Manner	High	275	49.8 / 46.4 / 48.1	0.0 / 0.0 / 0.0
	Low	59	23.7 / 48.3 / 31.8	0.0 / 0.0 / 0.0
	Neutral	7,301	97.7 / 97.6 / 97.6	100 / 95.6 / 97.8
	Macro Average	7,635	57.1 / 64.1 / 59.2	33.3 / 31.9 / 32.6
	Micro Average	7,635	95.4 / 95.4 / 95.4	95.6 / 95.6 / 95.6
Source	Current	7,510	99.1 / 99.2 / 99.2	100 / 98.4 / 99.2
	Other	125	53.6 / 50.0 / 51.7	0.0 / 0.0 / 0.0
	Macro Average	7,635	76.4 / 74.6 / 75.5	50.0 / 49.2 / 49.6
	Micro Average	7,635	98.4 / 98.4 / 98.4	98.4 / 98.4 / 98.4

A majority class-based baseline is shown for reference. (R)ecall/(P)recision/(F)score are reported.

The KT dimension has six values and, unlike other dimensions, it has no single majority value to which most events belong. The F-score for each value of this dimension is around 70%, except for the rare value *Method*, demonstrating that there are no extremely easy or difficult cases in the KT dimension. The performance of the CL dimension is lowest for the *L2* value. This seems reasonable, because the *L2* value is the middle value of this dimension, meaning that there is opportunity for such events to be misclassified as either of the other values in the dimension, i.e., *L1* and *L3*. In contrast, the performance of the system in predicting of the middle value in the Manner dimension, i.e., *Neutral*, is better than the prediction of the other values in this dimension. This is because the *Neutral* value is most frequently occurring value in this dimension. However, ambiguity with other values is still encountered during the the prediction of the *Neutral* value, as illustrated by the fact that the performance of the EventMine-MK system in predicting the *Neutral* value is lower than the baseline, in terms of F-score. The *Negative* value in the Polarity dimension and the *Other* value in the Source dimension are both quite rare values, which could be biased by their low frequencies. The detection of the *Other* value is the more difficult, since dictionary-based clue detection is more problematic for this value than for the *Negative* value. In the next section, however, we will show that machine learning-based clue detection can improve the performance.

### Extracting meta-knowledge enriched events

In Table 5, we summarise the results achieved by the EventMine-MK system trained on the ST-MK corpus. Since our aim here is not to evaluate the performance of event extraction *per se*, but rather to provide an intrinsic evaluation of the quality of the meta-knowledge assigned to these events, evaluation was only performed on those events whose complete structure (triggers and all arguments) was correctly recognised by the system. Of the 1,687 events in the ST test set, 1,017 events were correctly extracted by EventMine-MK. The results of two different experiments are shown in Table 5. The first experiment uses only features extracted from the ST-MK corpus (i.e., all features that were introduced in the *Meta-knowledge assignment system* subsection of *Methods*), while the second experiment (+GENIA) incorporates additional features from the meta-knowledge assignment model trained on the GENIA-MK corpus, as was explained in the section *Integrating the meta-knowledge assignment system with EventMine*. We employed both configurations described in the *Meta-knowledge assignment system* subsection of *Methods*, i.e., biased regularisation factors and type-based feature normalisation.

**Table 5:** Results of meta-knowledge assignment and event extraction for EventMine-MK trained on the ST-MK corpus

**Dimension**	**Value**	**Count**	**R / P / F**	**+GENIA (R/P/F)**	**Majority (R/P/F)**
KT	Investigation	38	76.3 / 67.4 / 71.6	68.4 / 65.0 / 66.7	0.0 / 0.0 / 0.0
	Analysis	132	70.5 / 69.9 / 70.2	72.7 / 73.3 / 73.0	0.0 / 0.0 / 0.0
	Observation	412	80.6 / 77.4 / 79.0	78.6 / 75.2 / 76.9	100.0 / 40.5 / 57.7
	Fact	59	33.9 / 76.9 / 47.1	40.7 / 70.6 / 51.6	0.0 / 0.0 / 0.0
	Method	8	0.0 / 0.0 / 0.0	0.0 / 0.0 / 0.0	0.0 / 0.0 / 0.0
	Other	368	77.4 / 73.8 / 75.6	76.6 / 74.0 / 75.3	0.0 / 0.0 / 0.0
	Macro Average	1,017	56.5 / 60.9 / 57.3	56.2 / 59.7 / 57.3	16.7 / 6.8 / 9.6
	Micro Average	1,017	74.6 / 74.6 / 74.6	73.9 / 73.9 / 73.9	40.5 / 40.5 / 40.5
CL	L3	983	97.7 / 98.9 / 98.3	98.6 / 99.0 / 98.8	100.0 / 96.7 / 98.3
	L2	30	73.3 / 48.9 / 58.7	76.7 / 62.2 / 68.7	0.0 / 0.0 / 0.0
	L1	4	0.0 / 0.0 / 0.0	25.0 / 100.0 / 40.0	0.0 / 0.0 / 0.0
	Macro Average	1,017	57.0 / 49.3 / 52.3	66.8 / 87.1 / 69.2	33.3 / 32.2 / 32.8
	Micro Average	1,017	96.6 / 96.6 / 96.6	97.7 / 97.7 / 97.7	96.7 / 96.7 / 96.7
Polarity	Positive	975	97.5 / 98.8 / 98.1	98.2 / 98.6 / 98.4	100.0 / 95.9 / 97.9
	Negative	42	71.4 / 55.6 / 62.5	66.7 / 60.9 / 63.6	0.0 / 0.0 / 0.0
	Macro Average	1,017	84.5 / 77.2 / 80.3	82.5 / 79.8 / 81.0	50.0 / 47.9 / 48.9
	Micro Average	1,017	96.4 / 96.4 / 96.4	96.9 / 96.9 / 96.9	95.9 / 95.9 / 95.9
Manner	High	56	78.6 / 63.8 / 70.4	76.8 / 65.2 / 70.5	0.0 / 0.0 / 0.0
	Low	4	100.0 / 66.7 / 80.0	100.0 / 66.7 / 80.0	0.0 / 0.0 / 0.0
	Neutral	957	97.2 / 98.7 / 97.9	97.4 / 98.6 / 98.0	100.0 / 94.1 / 97.0
	Macro Average	1,017	91.9 / 76.4 / 82.8	91.4 / 76.8 / 82.8	33.3 / 31.4 / 32.3
	Micro Average	1,017	96.2 / 96.2 / 96.2	96.3 / 96.3 / 96.3	94.1 / 94.1 / 94.1
Source	Current	1,003	99.8 / 99.5 / 99.7	99.9 / 99.5 / 99.7	100.0 / 98.6 / 99.3
	Other	14	64.3 / 81.8 / 72.0	64.3 / 90.0 / 75.0	0.0 / 0.0 / 0.0
	Macro Average	1,017	82.1 / 90.7 / 85.9	82.1 / 94.8 / 87.4	50.0 / 49.3 / 49.7
	Micro Average	1,017	99.3 / 99.3 / 99.3	99.4 / 99.4 / 99.4	98.6 / 98.6 / 98.6
Event (Task 1)		1,789	55.1 / 61.7 / 58.2		
Event (Task 2)		1,795	54.1 / 60.8 / 57.2		
	Event (Task 3)		1,997	51.6 / 59.4 / 55.3		

Meta-knowledge assignment performance is evaluated only on events correctly predicted by the system. Event extraction performance is evaluated using the ST evaluation tools. A majority class-based baseline is shown for reference. +GENIA shows the results obtained when the outputs of the GENIA-trained meta-knowledge assignment model are incorporated as additional features. (R)ecall/(P)recision/(F)score are reported.

Examining the results of the first experiment, differences in performance can be observed between the systems trained on the GENIA-MK corpus (Table 4) and the ST-MK corpus. For all dimensions, the micro-averaged scores on the ST-MK corpus are a few points better than those on the GENIA-MK corpus, but the differences in the macro-averaged scores are dependent on the dimensions. Whilst these differences can partly be attributed to the much smaller number of events in the ST-MK corpus (and hence less training data), they can also be attributed to differences in the distribution of meta-knowledge values between the two corpora, as illustrated in Table 2. In general, if meta-knowledge values appear comparatively less frequently in the ST-MK corpus than in the GENIA-MK corpus, then a degradation in performance can be observed in EventMine-MK, compared to the meta-knowledge assignment system trained on the GENIA-MK corpus. This is most notably the case for *L1* and *Method*, which appear less than half as frequently in the ST-MK corpus as in the GENIA-MK corpus. Because of this, EventMine-MK is unable to make any correct predictions for these meta-knowledge values. The same effect can be seen, but to a much lesser extent, with the meta-knowledge values *Analysis*, *Fact*, and *L2*, causing a drop in the macro-averaged F-scores the KT and CL dimensions, compared to the standalone meta-knowledge assignment system trained on the GENIA-MK corpus. In contrast, *Observation*, *High* and *Low* appeared more frequently as meta-knowledge values in the ST-MK corpus than in the GENIA-MK corpus, resulting in a large increase in ability of EventMine-MK to predict these values correctly, compared to the results shown in Table 4. Accordingly, the macro-averaged F-score for Manner also improved. There is little change in the performance of the prediction of the Polarity values, i.e., *Positive* and *Negative*, compared to Table 4, meaning that the macro-averaged F-score for Polarity did not alter very much. An exception to the general rule can be observed in the case of the *Other* value of the Source dimension. Although this appeared less often in the ST-MK corpus than in the GENIA-MK corpus, performance was improved in EventMine-MK. This was mainly due to the additional meta-knowledge clues extracted by the trigger/entity detector, which proved useful in comparison to the use of only dictionary match and citation features (regular expressions). If the additional meta-knowledge clues are not used, then performance drops from 75.6% to 47.6% F-score. This improvement in the prediction of the *Other* value resulted in a higher macro-averaged F-score for Source, compared to the results shown in Table 4.

According to the results of the second experiment, the addition of features from the meta-knowledge assignment model trained on the GENIA-MK corpus (+GENIA) improves the performance of the EventMine-MK trained on the ST-MK corpus, in terms of macro-averaged F-score. Although the results for the *Method* value of the KT dimension could not be improved, since this meta-knowledge value appears extremely rarely in the ST-MK corpus, performance on most other rare meta-knowledge values was improved by the use of these additional features. Direct application of the GENIA-trained meta-knowledge assignment model to the ST test set was also evaluated, but performance was reduced for all dimensions. This degradation in performance is due to the differences in the event types and in the distribution of meta-knowledge types between the two corpora. However, our results demonstrate that, when used indirectly, information from the GENIA-MK corpus can improve the recognition of meta-knowledge for the ST events.

We performed an error analysis of the results produced by the system that incorporates additional features from the GENIA-trained system.

KT has the highest number of meta-knowledge values, some of which need to be disambiguated according to their contexts. For most values of KT, some instances were misclassified as *Other*, especially if clue expressions were not present, since this is one of the most commonly occurring values. *Fact* instances were often misclassified as *Analysis* or *Observation*, since these two values appear more frequently than *Fact* and are sometimes ambiguous, in that they share a small number of clue expressions. Errors in *Method* occurred because the clue words were not detected, and furthermore, because the trigger words *(co)transfection*, which are present in all incorrectly classified *Method* events, occur far more frequently as trigger expressions for *Observation* than for *Method* in the training data sets. For CL, no instances were misclassified as *L1*. Three instances that should have been assigned *L1* were misclassified as *L3*, since *L1* clue words were not detected by the system. With regard to Polarity, errors often occurred due to negation of event arguments in coordination (e.g., *but not TNF-alpha*) and incorrect detection of nested events. Negation often attaches to the parent of the nested events, so if the nested structure is wrongly detected, the Polarity value of the argument event will be inverted. For example, if the gold-standard *regulation* event in the phrase *no regulation of phosphorylation* is annotated as negated, but the system only detects the *phosphorylation* event and not the *regulation* event, the *phosphorylation* event would be erroneously detected as being negated. Errors in Manner occurred between neighbouring meta-knowledge values (*High* and *Neutral*, and *Neutral* and *Low*), for reasons similar to the errors that occurred for the CL dimension. In terms of Source, some *Current* events were wrongly detected as *Other* when clue words for *Other* were detected in the same sentence. Conversely, some *Other* events were detected as *Current* when the clues were not detected by the system.

### Negated and speculated event extraction on the ST corpus

EventMine-MK was applied to the BioNLP’09 ST subtask (Task 3) of extracting events with associated negation and speculation information. This task does not deal with all the meta-knowledge dimensions that can be recognised by our system, but applying our system to this task is useful to allow comparison with other systems that can extract negated and speculated events. Two different versions of EventMine-MK were trained, one on the ST-MK corpus, and one on the original ST corpus, which was annotated for both negation and speculation, but not for negation and speculation clues. Using the ST-MK corpus, EventMine-MK is able to construct a meta-knowledge clue dictionary and a meta-knowledge clue detector. For the EventMine-MK version trained on the ST corpus, such functionality was not possible, given the lack of negation and speculation clues in this corpus. Table 6 shows the results and compares these with the scores of the top performing systems that participated in Task 3 of the ST. Performance is reasonably low for all the systems in this table, because the evaluation settings take into account event extraction performance as well as negation/speculation detection. As shown in Table 6, our novel systems (EventMine-MK with or without meta-knowledge clues) outperform the other systems compared, in terms of both overall F-score, and in terms of negation detection. In most cases, recall is also higher than for the other systems. The meta-knowledge clue annotation helps to improve performance, especially in the detection of negated events, for which a considerable improvement in performance can be observed over the version of the system that does not use clues. Conversely, for speculation, a small decrease in performance can be observed when meta-knowledge clues are taken into account. However, this decrease reinforces the analysis by [44] that speculation annotations in the ST corpus do not conform to the standardised notion of speculation, i.e., in contrast to the events enriched with meta-knowledge annotation, events occurring with modal verbs (e.g., *may*) and epistemic adverbs (e.g., *probably*) are rarely annotated as speculative in the ST corpus. According to this feature of the ST corpus, ignoring lexical clues increases the F-score according to the ST evaluation settings.

**Table 6:** Negated and speculated event extraction (Task 3) results on the ST corpus

	**Negation**	**Speculation**	**Total**
EventMine-MK(+clues)	**29.96** / 42.24 / **35.05**	21.63 / 36.59 / 27.19	**25.98** / 39.79 / **31.43**
EventMine-MK	28.19 / 36.16 / 31.68	22.12 / **41.82** / 28.93	25.29 / 38.33 / 30.47
[37]	22.03 / 49.02 / 30.40	19.23 / 38.46 / 25.64	20.69 / 43.69 / 28.08
[77]	18.06 / 46.59 / 26.03	**23.08** / 40.00 / **29.27**	20.46 / 42.79 / 27.68
[44]	15.86 / **50.74** / 24.17	14.98 / 50.75 / 23.13	16.83 / **50.72** / 25.27

Results are shown both with the use of meta-knowledge clues (+clues) and without the use of clues. Recall / Precision / F-score are shown for each value, and the highest scores are shown in bold.

### Analysis of meta-knowledge distribution on abstracts and full texts

To investigate the differences in the performance of our system on abstracts and full texts, we applied the model in Table 6 to the full-text subset of BioNLP-ST’11 GENIA corpus. Table 7 shows the performance of negated and speculated event extraction, using meta-knowledge clues, on the full-text subset. This table shows that the performance of EventMine-MK is almost consistent on both abstracts (the ST corpus) and full texts, and that the EventMine-MK system outperforms other systems on the full texts. It should be noted that EventMine-MK system used only the abstracts (the ST-MK corpus) for training, unlike the other systems shown, which used both abstracts and full texts for training purposes.

**Table 7:** Negated and speculated event extraction results on the full-text subset of the BioNLP-ST’11 GENIA corpus

	**Negation**	**Speculation**	**Total**
EventMine-MK	**34.85** / 40.00 / **37.25**	**19.00** / **38.00** / **25.33**	**25.30** / **39.00** / **30.69**
[77]	21.21 / 38.24 / 27.29	17.00 / 34.69 / 22.82	18.67 / 36.14 / 24.63
[37]	25.76 / **48.28** / 33.59	15.00 / 23.08 / 18.18	19.28 / 30.85 / 23.73

EventMine-MK used the model with meta-knowledge clues in Table 6. Recall / Precision / F-score are shown for each value, and the highest scores are shown in bold.

EventMine-MK was then trained on the ST-MK training and development sets, and applied to the ST test set and the full-text subset of BioNLP-ST’11 GENIA corpus, in order to compare the meta-knowledge distribution in abstracts and full texts. In this experiment, additional features from the meta-knowledge assignment model trained on the GENIA-MK corpus (as explained earlier) were incorporated. Table 8 compares the distributions of meta-knowledge assignments in the abstracts and full texts. Whilst it should be noted that the results obtained for full papers may not be completely accurate, given that full papers (whose characteristics are different from abstracts) did not feature at all in the training data for the system, some interesting trends can nevertheless be observed. The statistics shown in Table 8 suggest that full texts tend to include more events denoting previous work (*Other*), more speculated events (*L1* and *L2*), more events denoting *Low* manner, and fewer events denoting general facts (*Fact*) than abstracts. In order to confirm these general trends, and to help to improve the accuracy of the automatic assignment of meta-knowledge to events in full papers, we intend to manually enrich the full papers released as part of the BioNLP-ST’11 GENIA corpus with meta-knowledge as future work. The enriched event annotations in the papers can subsequently be used as training and test data to help to improve and evaluate the performance of EventMine-MK on full papers.

**Table 8:** Distribution of instances by frequency and percentage (%) for each meta-knowledge value

**Value**	**ST**		**FullText**		**FullText(%)/ST(%)**
	**frequency**	**%**	**frequency**	**%**	
Investigation	131	5.2	201	5.8	1.11
Analysis	312	12.4	477	13.7	1.10
Observation	1,154	45.8	1,465	42.0	0.92
Fact	89	3.5	94	2.7	0.76
Method	0	0.0	4	0.1	N/A
Other	832	33.0	1,244	35.7	1.08
L3	2,415	95.9	3,283	94.2	0.98
L2	87	3.5	162	4.6	1.35
L1	16	0.6	40	1.1	1.81
Positive	2,389	94.9	3,294	94.5	1.00
Negative	129	5.1	191	5.5	1.07
High	165	6.6	241	6.9	1.06
Low	18	0.7	39	1.1	1.57
Neutral	2,335	92.7	3,205	92.0	0.99
Current	2,487	98.8	3,415	98.0	0.99
Other	31	1.2	70	2.0	1.63
Event Total	2,518	100.0	3,485	1.0	100.00

Distributions of predictions on test set of the ST corpus and on the full-text subset of the BioNLP-ST’11 GENIA corpus are reported. EventMine-MK used the model trained on the ST-MK corpus with the +GENIA setting, as in Table 5. The last column shows the ratio of the percentage in the full-text subset to the percentage in test set of the ST corpus.

## Conclusions

We have presented a novel system that can extract biomedical events from the literature and assign meta-knowledge to them. The system was constructed by integrating a new meta-knowledge assignment system into a state-of-the-art event extraction system, EventMine. The meta-knowledge assignment system was firstly evaluated on the GENIA-MK corpus, on which it performed well compared to the baseline, with a small number of exceptions. This assignment system was then integrated in EventMine. The augmented version of EventMine, which we call EventMine-MK, was trained on the ST-MK corpus, to which meta-knowledge annotation had been transferred from the GENIA-MK corpus. With the help of features from the model trained on the GENIA-MK corpus, EventMine-MK was able to assign meta-knowledge to the detected events with a good degree of accuracy, comparable to the performance of the meta-knowledge assignment system trained on the GENIA-MK corpus. EventMine-MK was also able to outperform other state-of-the-art event extraction systems in the task of detecting negated and speculated events. EventMine-MK is available as a UIMA component, which is available within the U-Compare interoperable text mining system.

As future work, we will apply EventMine-MK to the entire PubMed abstract collection and thus be able to extract events with meta-knowledge for use by common domain applications. We ran EventMine-MK with no parallelisation on a cluster with 32 Intel(R) Xeon(R) X7560 (2.27GHz) CPUs with 330 GB RAM using hyper-threading. On average, it took 5.7 seconds to extract events with meta-knowledge assignment from a single abstract, of which an average of 2.1 seconds was used for meta-knowledge assignment. It will take approximately 307 CPU days to apply meta-knowledge assignment to the existing event extraction results for the entire PubMed abstract collection, which includes about 12 million abstracts and 9 million titles. Such meta-knowledge can be used to rerank and/or restrict search results in semantic search engines, e.g., the MEDIE intelligent search system [47], and to construct pathways that use only factual or trusted events. Crucially, such fine-grained information about events is important for semantic publishing, e.g., SciVerse [99], and semantic web applications, e.g., Open PHACTS [100]. Our approach creates living texts, which can be viewed in a number of different ways to extract assertions, hypotheses, contradictions, negations, etc. We also wish to improve the performance of the meta-knowledge assignment, by finding a way to treat the dependencies among values and relations between meta-annotation clues and events.

## Competing interests

The authors declare that they have no competing interests.

## Author’s contributions

All authors contributed to the production of the manuscript. PT, JM and SA supervised all steps of the work. MM built the system and carried out the experiments. All authors read and approved the final manuscript.
